# Mobile Emergency, an Emergency Support System for Hospitals in Mobile Devices: Pilot Study

**DOI:** 10.2196/resprot.2293

**Published:** 2013-05-23

**Authors:** Pierfrancesco Bellini, Sergio Boncinelli, Francesco Grossi, Marco Mangini, Paolo Nesi, Leonardo Sequi

**Affiliations:** ^1^University of FlorenceDepartment of Information EngineeringFlorenceItaly; ^2^Careggi University Hospital, Florence, ItalyFirenzeItaly

**Keywords:** emergency, hospital emergency, emergency communication management, mobile emergency

## Abstract

**Background:**

Hospitals are vulnerable to natural disasters, man-made disasters, and mass causalities events. Within a short time, hospitals must provide care to large numbers of casualties in any damaged infrastructure, despite great personnel risk, inadequate communications, and limited resources. Communications are one of the most common challenges and drawbacks during in-hospital emergencies. Emergency difficulties in communicating with personnel and other agencies are mentioned in literature. At the moment of emergency inception and in the earliest emergency phases, the data regarding the true nature of the incidents are often inaccurate. The real needs and conditions are not yet clear, hospital personnel are neither efficiently coordinated nor informed on the real available resources. Information and communication technology solutions in health care turned out to have a great positive impact both on daily working practice and situations.

**Objective:**

The objective of this paper was to find a solution that addresses the aspects of communicating among medical personnel, formalizing the modalities and protocols and the information to guide the medical personnel during emergency conditions with a support of a Central Station (command center) to cope with emergency management and best practice network to produce and distribute intelligent content made available in the mobile devices of the medical personnel. The aim was to reduce the time needed to react and to cope with emergency organization, while facilitating communications.

**Methods:**

The solution has been realized by formalizing the scenarios, extracting, and identifying the requirements by using formal methods based on unified modeling language (UML). The system and was developed using mobile programming under iOS Apple and PHP: Hypertext Preprocessor My Structured Query Language (PHP MySQL). Formal questionnaires and time sheets were used for testing and validation, and a control group was used in order to estimate the reduction of time needed to cope with emergency cases. First, we have tested the usability and the functionalities of the solution proposed, then a real trial was performed to assess the reduction in communication time and the efficiency of the solution with respect to a case without Mobile Emergency tools.

**Results:**

The solution was based on the development of a mobile emergency application and corresponding server device to cope with emergencies and facilitate all the related activities and communications, such as marking the position, contacting people, and recovering the exits information. The solution has been successfully tested within the Careggi Hospital, the largest medical infrastructure in Florence and Tuscany area in Italy, thus demonstrating the validity of the identified modalities, procedures, and the reduction in the time needed to cope with the emergency conditions. The trial was not registered as the test was conducted in realistic but simulated emergency conditions.

**Conclusions:**

By analyzing the requirements for developing a mobile app, and specifically the functionalities, codes, and design of the Mobile Emergency app, we have revealed the real advantages of using mobile emergency solutions compared to other more traditional solutions to effectively handle emergency situations in hospital settings.

## Introduction

Disaster response to in-hospital mass-casualty incidents represents one of the greatest challenges in emergency management. Hospitals must provide care to large numbers of casualties in damaged infrastructures, with personal risks, limited resources, inadequate communications, and lack of information [1]. Often, data regarding the true nature of the incident are inaccurate, needs are not clear, hospital personnel is not efficiently coordinated nor informed on the real available resources. In this chaotic environment, new technologies in communications and advanced "smart devices" have the potential to vastly improve the emergency medical response to incident disasters. There are numerous examples of the benefits of timely access to information in emergencies and disasters. The role of information technology is becoming increasingly important for information-sharing during emergencies and disasters, including sensible information and video [2]. Most reports of information technology applications to emergencies or disasters concern applications that are hospital-based or occur during non-response phases of events [3-5]. In most cases, the information is propagated via voice [6], while it is also accepted that relevant information has to be communicated via chat or messaging. Also, logistics were noted to be of concern, in particular regarding the movement of personnel and patients within and outside the hospital. The availability of a mobile device granting support in locating hospital personnel and emergency exits can be of great help. There is consensus that information and communication technologies can play a vital role in coordinating crisis response between pre-hospital services and the hospital emergency departments.

In particular, new-generation mobiles devices with wireless Internet, television-camera, and geo-positioning may have the greatest impact on improving communications, information management and distribution, the overall disaster response, and the emergency medical care [3]. Medical personnel need to access updated information and knowledge in emergency conditions when staff is demanded to cover different roles. This information allows medical and paramedical personnel to adopt local standard intervention protocols and prescribe appropriate pharmaceutical dosages based on specific patient conditions during continuously changing situations. In hospitals, continuously updated information on protocols and prescription dosages is propagated in short time or real-time through specific terminals and mobile devices especially in emergency/critical-conditions.

Therefore, mobile devices are mandatory tools for information access and to help in decision making. On such grounds, the solution has to guarantee the access to any right and updated information in the needed time [7-10]. Physicians found the usage of personal device assistants (PDAs), which are comparable to smartphones, very useful during night duty and in emergency conditions [11]. Medical personnel tested intelligent, triage-based PDA systems that can gather all the emergency medical services with positive results [12].

The purpose of this study was to test an emergency system solution, called Mobile Emergency, which was designed to improve the readiness of hospital personnel during emergencies and allowing more efficient treatment procedures to be performed to the victims of disasters. The mobile application can help the hospital personnel to communicate with the in-hospital emergency headquarter, also known as the Central Station, to obtain better information on the situation, resulting in the best possible care for patients.

The main idea behind Mobile Emergency was to provide a support for managing communications among medical personnel during maxi emergencies that may occur into large medical centers. Large medical centers are made of several buildings located in a large area. The medical center is a sort of village where thousands of medical personnel units work with thousands of patients and visitors on any given day. In these large and complex scenarios, several emergency events may occur per week, and at times per day. They may range from simple water problems (flooding or shortages), lack of power, problems on oxygen, to serious fire outbursts. Moreover, in some cases the emergency may arrive externally from disasters (eg, an earthquake, a big crash in the railway/highway, a terrorist attack, a gas explosion in the city, etc), thus forcing a localized reorganization within the hospital to cope with an increasing stream of people and patients. These events may cause an unusually high number of new patients entering the hospital emergency area and affecting other specialized units (eg, burn units), thus snarling up the reception desk structure and analysis centers. Among the possible internal emergencies, fire is one of the most awkward situations as it may require general evacuation of hospital patients and staff, taking patients away from their care. Our aim was to create an application for mobile devices to improve the readiness of hospital personnel during emergencies, facilitate communication, assure positioning, provide information and knowledge, and help rescue teams in taking action, thus allowing more efficient rescue operations for the victims.

In hospitals and during emergency medical situations, there are many additional constraints. In general, communication connections (ie, WiFi; universal mobile telecommunications system, UMTS; general packet radio service, GPRS) can be discontinuous when patients are moved along passageways, in the countryside, in tunnels, on the street, or on the ambulance. In this scenario, off-line services on mobile devices may not be powerful enough, as information and knowledge is no longer accessible in real-time. The ideal mobile device should to be able to foresee the user’s intentions and wishes, and be capable of providing suggestions within the context of the situation and user profile. The information has to be recovered and processed intelligently to provide suitable suggestions to medical personnel in real-time. In the context of this paper [10], this is the so-called Mobile Medicine scenario.

This paper describes the main scenarios and requirements for the mobile emergency solutions. The architecture of the mobile emergency solutions and details of the proposed mobile app Mobile Emergency are also described. The coding of the quick response (QR) code for location modeling is presented, together with some snapshots of the mobile application. The Mobile Emergency tools used during operative conditions and data on the validation experiments are depicted in the Results section, together with critical and positive comments and opinions collected during the validation phases of the trials.

## Methods

### Phase 1: Requirements Analysis

In order to extract and identify the requirements for developing this mobile app, the general scenarios regarding the emergency conditions have been formalized and depicted by using standard unified modeling language (UML) models. They have been revised with the experts about maxi emergency completing them with all needed details and explaining the critical aspects about the communication. Analyzing the scenarios allowed us to identify a number of requirements, which we classified into 2 groups: (1) requirements collected according to the point of view of the medical personnel, and (2) general level requirements which included the point of view of the Central Station monitoring the disaster and supporting, coordinating the personnel, and the needed intervention actions. The Central Station is typically in the central service room (in the hospital administration building) to manage emergency in the hospital.

Finally the requirements were extracted and formalized. The identification of the requirements was performed by reviewing the scenarios, reviewing the literature, and interviewing emergency experts and various medical personnel within the hospital area, asking them to answer questions regarding what is needed, what should be avoided, and how the issues are evolving in the best and worst cases.

### Phase 2: Design and Development

The Mobile Emergency app can be installed on an iPad, iPhone, or iPod to provide the medical personnel with the needed support during the emergency. The Mobile Emergency app is distributed on Apple Store free of charge. The Mobile Emergency app provides a user-friendly interface and medical personnel do not need to spend too much time and effort on learning how it works. It was designed according to the ISO 13407:1999 standard, which deals with user centered development aspects. Our user centered development process started with the definition of the scenarios and requirements, and their validation with referent users. The next phase was the effective coding for development of a first prototype that has been tested and validated by previous and other users. The process itself is typically iterative, grounded on macro and micro life cycles. Macro lifecycle are those in which major features have to be analyzed, developed, and tested. They are planned to have a duration of 1-2 months. Micro cycles cope with the single elementary aspects of a feature development and have typically a duration of 1-2 weeks, including detailed analysis, development, and validation. The application was written using Objective-C and SQLite was used for data storage. The server component was written in PHP/HTML using an SQL server for data storage.

### Phase 3: Test and Validation

The proposed solution has been exploited during validation trials. The test and validation has been performed by following 2 phases. First, we tested the usability of the functionalities of the solution proposed, and second, a real trial was performed in order to assess the reduction in communication time and the efficiency of the solution with respect to case without Mobile Emergency tools. The assessment of the usability of the functionalities of the solution proposed was performed after 10 minutes with the mobile phone in the hands of the participants, describing what can be done and how. Such tests have been performed in the small area of the Careggi University Hospital, which is a large community hospital located in Florence, Italy. The hospital has approximately 100 different buildings, connected with streets and underground tunnels. Each building may have thousands of rooms, elevators, stairs, corridors, telephone numbers, cafeterias, restrooms, and local receptions.

In order to verify the validity of the solution, 30 medical personnel with previous experience in dealing with emergency events using conventional ways were included in the tests. A control group with 24 medical personnel were also involved with the same critical emergency conditions, but without the usage of the Mobile Emergency solution. This control group used mainly phone calls and maps hung on the walls of the hospital when possible. Both groups were not familiar with the paths to the facility’s exits from the critical area or how to reach the collecting areas. In addition to the participants, there was an observer and an evaluator. The emergencies consisted of fire, smoke, in single and multiple rooms, with the corridor also shrouded in smoke. The participants of the experimental group were asked to follow the instructions provided by the Mobile Emergency app. A variable number of patients were involved as well, with some visitors that did not abide by the decisions of the medical personnel. One of the tasks that participants had to perform was to move two patients from emergency situations. The personnel were asked to form groups in order to move two patients. One patient was in a room where the ceiling was about to give way, while the other patient was trapped in a fire in part of the building. The described trials have been replicated twice with different people (both patients and participants). The same identical conditions and commitments were asked to the control groups.

In order to assess the validity and effectiveness of the mobile solution for emergency situations within the hospital, the following indicators were taken into account: the estimation of the reaction time with and without using the Mobile Emergency solution, evaluation of clarity about the leadership in the participants groups (in both cases, validation and control), the speed to reach the exit, the usability of the mobile app in presence of fire and smoke (different levels), the effect of different degrees of illumination on use, presence and absence of network connection, and different size of the QR code.

## Results

### Identified Requirements

The medical personnel (doctors and nurses) are typically the first people to inform the Central Station about an emergency condition. According to most emergency protocols, the alarm may be sent by a phone call, an SMS, or other mechanisms. In most cases, additional information is needed to identify and assess the emergency conditions and the people at risk. An image and/or a video depicting the emergency event immediately at its occurrence could be useful (for better understanding and for reviewing later the conditions).

During an emergency, the medical personnel and the hospital have to be reorganized to provide patients with assistance. Many personnel are required to evacuate patients, for example, 4-6 medical personnel are needed to evacuate a patient confined to a bed. The traditional emergency guidelines and protocols do not offer support for team creation to cope with such problems. Furthermore, additional gathering areas for each emergency triage level have to be set up. These activities may be performed by recalling in a medical personnel temporarily from other areas of the hospital, thus permitting direct communication among personnel.

If the recalled medical personnel have to keep abreast of the specific situation with updates on the procedures and tools to be applied during that emergency, the hospital organization has to provide them with the missing information. For example, with tutorials, checklists, dosages tools, decision support systems [10]. To make this information more accessible, checklists on the activity to be performed for different emergency situations should be available on mobile phones. Moreover, such devices have to be continuously updated since many involved aspects may change.

The personnel involved in the emergency may be not fully aware about the precise location of each department, and building. Therefore, they have to be supported in finding the rooms and departments where their presence is requested, for example to help other personnel to cope with patients confined to bed or for other critical situations.

If the position of each medical personnel within the hospital is made accessible, the Central Station may better coordinate the reaction to the emergency event. Central administration could provide a device or tag for each medical personnel to allow such tracking. Although constant position tracking of medical personnel may be a privacy violation, their position may be inquired anyway by Central Station when called for support at the emergency scene. There are many technical solutions to identify positions, including WiFi, radio-frequency identification (RFID), global positioning system (GPS), QR code, and maps (typically outdoor maps, as Google Maps, TomTom). WiFi might not be the best solution for all cases, as it is not very precise and dependent on power. RFID readers are not available on low cost mobile devices (they could be used to read the passive RFID tags reporting location information). GPS solutions have low reliability in underground tunnels and within buildings. QR codes are very simple and cheap; they can be placed inside several stable elements such as plates, indication printouts, and maps. This means they can be integrated very easily with current widespread solutions, which are based on maps and plates. In emergency situations, the medical personnel and staff at the Central Station need to react in certain ways, outlined in Textboxes 1 and 2.

**Considerations of medical personnel in emergency situations.**

Provide information regarding the emergency inception to the Central Station. It can be performed by using a simple form (to collect the minimal formal information, such as the situation and its gravity) or in a more effective manner by providing a picture or a video describing the detected emergency conditions.
Recover the position within the hospital and obtain the easiest, feasible, and updated path to exit from the area interested by the emergency. The best exit path may change over time according to the changing emergency conditions (eg, if a certain set of stairs to an emergency route is blocked), thus the medical personnel has to keep abreast of the situation and updates are needed according to the patients’ conditions and disabilities.
Communicate the identified position to the Central Station to receive help and make easier for the Central Station to gather medical personnel for the evacuation.
Get in contact with neighboring medical personnel to receive help in moving a patient or support to operate on a patient. The system should be able to identify the nearby colleagues and provide the means of contacting them.
Recover information about the emergency collecting areas for patients who may share triage tags or other colored standard labeling. The paths to reach the collecting areas are also very relevant; in most cases, it is not enough to have one of the exits, but to have the correct one that may bring you to the specific collecting area where your patients have to be led to.
Recover information about the emergency severity, status updates, and whether people in the area are in danger, to arrange for an evacuation. In most cases, the best solution to cope with the emergency is to wait for its close without abandoning the room where the emergency managing personnel recommended. When it becomes difficult to communicate the best possible solution for a specific emergency, the fastest and easiest way to communicate would be via direct mobile messaging using push notification.
Recover the position of the emergency coordinator and the related team to cope with specific intervention fields, such as the transfer of a patient, the management of a collecting area, etc.
Recover and/or get access to procedures to be followed such as: ACLS, BLS, and/or checklists, decision support, dosages to be applied, etc [10]. This information is typically available in the central room of the medical department and may not be accessible from the location of the medical personnel at the time, for example in a patient room, as they cannot abandon their position in emergency conditions.

**Additional requirements of the emergency management via central station.**

Receive the emergency calls from mobile phones and other devices with the minimal but correct information. Such calls have to be collected on the basis of the local emergency manual or guidelines. Emergency conditions are coded according to their severity, measured in terms of the number of patient and/or people involved, their autonomy in moving, etc.
Aggregate the emergency calls coming from the same area and regarding the same emergency. This allows for a better evaluation of the severity of the emergency.
Keep track of the emergency status and its evolution, from its inception to its solution, and thus keeping the involved personnel informed on such changes. A trace record should be kept for the emergency event, from its inception, its confirmed status, and up to the final solution. The record should also contain information about the personnel involved, the performed actions, the involved rooms and departments, the temporal evolution, the involved patients, and other relevant details.
Identify medical personnel needing support in the emergency, support them in creating collaborative teams/groups and defining their coordinator, and/or reaching the collecting areas via escape doors.
Control the conditions of the medical personnel being potentially involved. In some cases, the Central Station of the hospital does not know precisely who is in the area under emergency. Personnel who are supposed to be in one area may not actually be in that area at the exact time of the emergency. To this end, a verification could be very useful to see if there are some missing people or unclear situations.
Identify medical personnel who could be involved in solving the emergency, since they are near the emergency location or are supposed to be in that area. In this case, the system should be able to alert them in order to communicate their condition and position to the Central Station.
Identify medical personnel who could be recalled to support other colleagues in managing problems and patients in the collecting areas. In this case, the system should be able to alert them while providing all the needed information, such as where to go and the contact point to be in touch with when they arrive. The selection of personnel to be recalled can be performed on a general basis or according to their position and/or competence profile.
Inform the personnel if patient evacuation is needed or if new incoming patients are arriving in the collecting areas.
Inform other hospitals and other institutions about the occurrence of the emergency, for example the fire department, the police, the Engineer Corps, and the military authorities.
Accept alarms that are coming only from qualified personnel. Therefore, the qualification cannot be limited to mobile devices, since it may be in the hands of unqualified people. On such grounds, authenticated access to emergency services is needed. Non-authenticated emergency calls and alarms can be accepted as well, but should be treated in a different manner.

### System Design and Development

**Figure 1:**
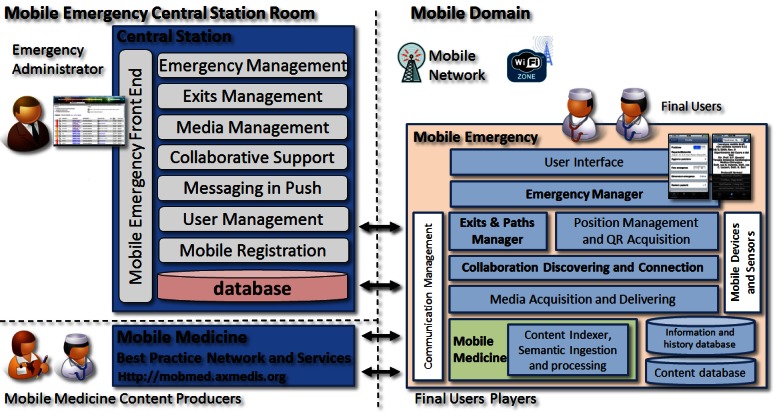
Architecture of the Mobile Emergency app: Central Station (left) and Mobile Emergency Application (right).

According to the above requirements, a Central Station and a Mobile Emergency app have been designed and developed. The design of both solutions used standard UML. Therefore, the main architecture of Mobile Emergency solution is composed of 3 main elements, depicted in Figure 1. The Mobile Medicine Best Practice Network and Service [10] is a service portal used to produce collaborative content for supporting the medical personnel during the emergency, in day-by-day conditions and for permanent medical education. The Best Practice Network is supported by a player application called Mobile Medicine, which has been taken as a starting point to develop Mobile Emergency solutions and mobile application [10]. The content itself may perform reasoning on semantics, thus providing different behaviors on the basis of content descriptors, user preferences, and contextual information [13]. The early version of Mobile Medicine was developed for PDA in Windows Mobile 6.5 and for iPhone by using MPEG-21 [14,15]. The present extended version of the Mobile Medicine application is called Content Organizer, available for iPad, iPhone, Windows Phone 7, and Android. Content Organizer served as the basis for further Mobile Emergency app development for these platforms.

The Central Station provides services to the Mobile Emergency apps, collecting emergency alarms, supporting the personnel during the emergency, providing support to identify the best escape doors, providing support to build collaboration among personnel, and sending messages to mobile devices by using the Apple Push Notification Service.

The main elements of Mobile Emergency app are marked in bold in Figure 1 (right). The permanent memory in the mobile phone is used to store the information regarding the maps (that are updated continuously), to store the procedure to be played by the Mobile Medicine tools, and to store the history of all the actions. These can be used to reconstruct possible problems and events whenever a legal analysis of the facts is requested. In respect of privacy policies, the user is informed about these aspects. The configuration of the system also includes the emergency protocol and classification according to the hospital emergency manual. This information is enforced into the system during the set up and configuration by using XML files.

The Emergency Manager allows the user to formalize and send the emergency alarms, with a media file attached (typically a video or some images, collected by using the Media Acquisition and Delivering module). The Emergency Manager verifies and follows the emergency manual adopted by the structure. For example, in the case of the Careggi Hospital, the emergency manual provides the emergency, inform the Central Station, the fire department, and transportation service, coordinate the first aid, coordinate the patients’ escape, verify if the alarm has been propagated, and inform the director. Moreover, the Emergency Manager monitors the status of the actual emergencies that have been signaled to and from the Central Server, and recovers all the communications and actions carried out on the device for possible reconstruction of the events and the performed actions. The Emergency Manager periodically gets fresh information about the emergency status, maps for exits and collecting areas, and may receive direct calls from the Central Server in push (by using the push service of Apple). The pushed information may include suggestions and assignments to move towards a collecting area, to join a team, to become the leader of a team, or to move to a different area and room.

Collaboration Discovering and Connection module allows the user to discover if there are some other colleagues in the nearby area according to the user’s position. If so, such colleagues are listed and their related positions stored and visualized in the present map. This allows the reception of direct calls from colleagues in the same emergency area. In certain conditions, it may be possible to have such communication carried out in spite of any lack of connection with the Central Station.

**Figure 2:**
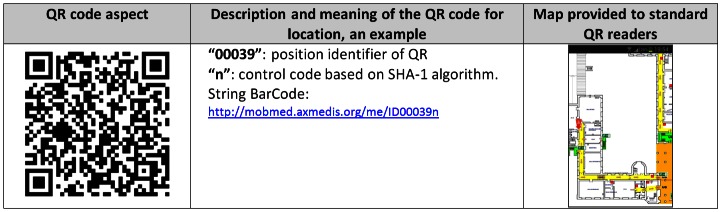
Example of QR coding regarding position.

The Exits and Paths Manager help the users to get their position via nearby QR codes (see Figure 2). QR code images are directly generated by the Central Server to be placed at any strategic points within the hospital. Once the user has taken his/her position, the device informs the Central Station and downloads the updated possible exit solution. In case of connection loss, the mobile device uses the maps stored into the local database to provide suggestions for directions. It finds the correct direction to get to the exit according to the emergency conditions and the locations of exits or collecting areas established by the Central Server. It also receives automatic map updates and other information.

The Exits and Paths Manager performs some reasoning about the map information/descriptor, taking into account the position and the movements of the users. It estimates the current position by using the accelerometers of the mobile device to perform adjustments with respect to the position set using a QR code or via GPS (for example when the user is outside). QR codes are coded with 30% redundancy where the QR string is defined as: <serverURL>ID<Position ID><checkdigit> (see Figure 2 left and middle). On the Mobile Emergency app, when the user has acquired the QR code, the device creates a connection to the Central Station by using the HTTP protocol, which provides the information of the corresponding position. If the user is authenticated, the system updates his/her position by inserting the new location of the user in the Central Station database. The access to QR code URL by means of the Mobile Emergency app implies the access to additional information used by navigation system, including: building code, department code, currently updated image URL, room code, spatial coordinates of the QR code position on the map, spatial coordinates of the nearest exits, and spatial coordinates of the nearest collecting areas. The Mobile Emergency app exploits this additional information and, together with the maps downloaded from the server, is able to navigate the user to the nearest exit, a collecting area, or a specific position.

On the other hand, the coding of a QR code as an URL allows the Central Server to react to different QR readers in a different manner. Thus, if the QR reader is not read by the Mobile Emergency Application, a simple map with the current position, exits, and collecting areas is provided (see Figure 2, right). This allows all users to exploit the information associated with QR code placed in the hospital by simple applications, even if with limited capabilities when there is no navigation, no emergency status, or no networking.

Mobile Medicine engine and mobile applications can download, store, retrieve, and put in execution Mobile Medicine intelligent content [10]. This content is meant for the information and the training education of the medical personnel. It can be recovered and downloaded by using a different model of QR codes.

The content item area is indexed according to the descriptors and taxonomical classification of medicine, and it provides support for querying and organizing content according to the user data and requests. The adoption of HL7 (Health Level Seven International) compliant protocols for communicating with general hospital information system should be performed inside the mobile medicine intelligent content [10]. These aspects are not central for the Mobile Emergency app and solution. Moreover, on the same mobile device, it is possible to have other medical applications addressing the access to the HL7 information as well. For these reasons, the other aspects of HL7 have not been addressed.

### System Test and Validation

**Figure 3:**
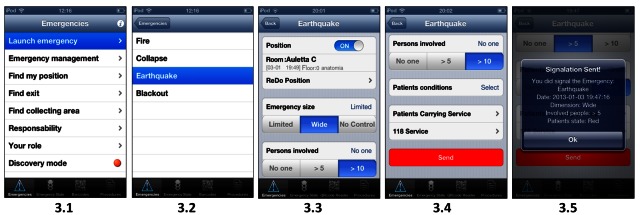
Scenario A: the Emergency Call delivering.

**Figure 4:**
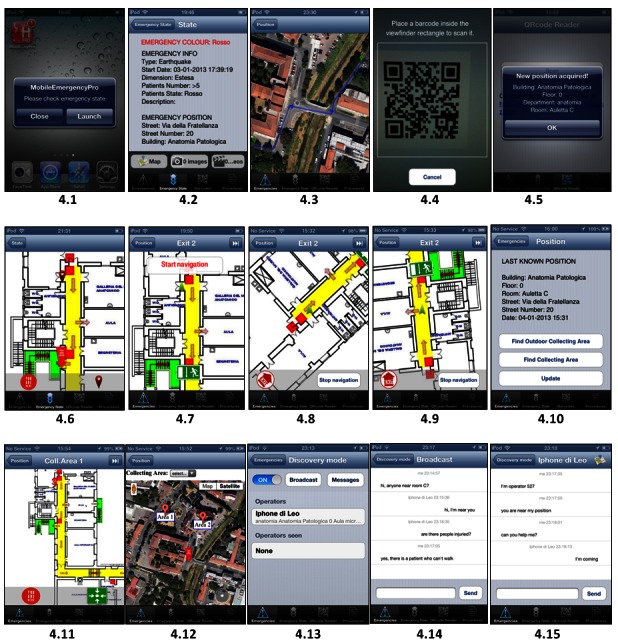
Scenario B: from 4.1-4.2, the emergency team reaches the emergency location. Scenario C: direct exit from (4.4-4.5) to move out (4.7-4.9). Scenario D: from emergency location (4.4-4.5) to collecting areas indoor (4.11) and outdoor (4.12). Scenario E shows some snapshots of direct discovering and chat among personnel in the emergency area (4.13-4.15).

A typical usage of the mobile application is depicted in Figures 3 and 4 through a series of scenarios. For more information about application use, consult the user guide.

The Scenarios A starts with an earthquake causing serious damage to the pathologic anatomy building. A medical operator uses the Mobile Emergency application and selects “Launch emergency” (3.1), the type of emergency (3.2), and the position, number, and the state of the people involved (3.3 and 3.4). Finally, the emergency is sent (3.4) and a confirmation is requested (3.5).

The personnel at the Central Station receives the emergency call and decides to send a rescue team (Scenario B). To this end, in Figure 4 (4.1 to 4.6), a push message is sent to rescue personnel to cope with the emergency (4.1) asking each of them to accept the message from Mobile Emergency app to check the status. Once the notification is accepted, the application displays the details of the emergency intervention (4.2). From that page, the user can select a map to get directions to the location where the emergency has occurred from outside or inside the building (4.3).

Once the right building has been spotted, the personnel can compare their position at the time with respect to the position of the emergency by using a QR code placed near doors and other relevant points (eg, maps on walls, 4.4 and 4.5). Once the position of the medical personnel is recognized, they can track their position on the building map in relation to the emergency location (4.6). The scenario can go on by reaching the point and addressing the emergency, performing the triage, and going to the exit (see Scenarios C) or moving the patients to the collecting area (see Scenario D). Scenario C describes the simpler point of view of personnel able to leave the emergency location without waiting for the rescue team. This scenario is depicted by considering the sequence (4.4) and (4.5) to take their own position and continue with (4.7) to (4.9) to reach Exit 2 assisted by the application’s internal navigator. Scenario D consists of moving from the emergency location to a collecting area. In those cases, the position can be taken by GPS or by using (4.4)-(4.5), while different maps can be selected by using (4.10) for reaching indoor (4.11) and outdoor (4.12) collecting areas.

Scenario E (in Figure 4) depicts the advantage of the local wireless exploitation in connecting the operators who can locate one another by using the discovery mode (4.12). With this functionality, operators can communicate both with a chat in broadcasting mode (4.13) and directly with a private chat with a single user (4.14).

### Mobile Medicine, Medical Procedures, and Content

In the Mobile Medicine scenario, the useful content types may range from *single files* (ie, audio, video, images, documents, slides, and animations) to *cross media files* (ie, files containing interactive supportive tools such as calculators of health measures to help the user make correct decisions). Examples are treatment algorithm triage, Assessment of Consciousness, SNG (Nasogastric tube), and Apache Score [10]. For example, the estimation about the probability of pulmonary emboli, the estimation of a dosage on the basis of patient weight, and the assessment of neurological conditions on the basis of standard quantitative models.

### Test and Validation: Numerical Data

The average time to perform the emergency call (Scenario A) was reduced by 18% (from an average of 57.33 seconds with SD 6.8 seconds, to 48.67 seconds and SD 2.3 seconds), with a higher value of the emergency call quality in terms of data (ie, precision of the emergency location and no missing information, see Table 1). According to Scenario B, once the directions from the Central Station had been received, the rescue team without help from the mobile app started to search for route to the emergency scene more quickly, whereas those with the mobile app spent more time on studying the map first before starting to move. After reaching the emergency location, triage was performed and the patients were moved to the same collecting area (Scenario D). Although medical personnel without the mobile app started to move faster, they took longer to reach the emergency area. Therefore, the total time for all movements (from the start time to the time they reached the emergency scene, but without the time spent on triage which was almost constant for all) showed that the groups with the Mobile Emergency app were much faster (mean time 9 minutes 57 seconds and SD 57 seconds) than those without (mean time 16 minutes 20 seconds and SD 7 minutes 5 seconds). This implies an averaged reduction in the time needed to move into the collecting area of more than 35%.

**Table 1:** Measured data from the experiments.

	With Mobile Emergency app		Without Mobile Emergency app	
	Average value	SD	Average value	SD
Time to perform the emergency call, sec	48.67	2.30	57.33	6.80
Quality of the emergency call in terms of received information, score^a^	4.98	0.05	3.66	0.22
Time to start going towards emergency location, min:sec	1:58	0:07	1:02	0:15
Time spent to reach the Emergency area by the rescue team, min:sec	4:19	0:34	12:58	6:33
Cumulated Time to perform all the movements from the beginning to the emergency location and from there to reach the collecting areas (excluding time to triage), min:sec	9:57	0:57	16:20	7:05

^a^The score was based on a Likert scale: 5=very good; 4=good; 3=barely acceptable; 2=poor; 1=very poor.

## Discussion

The results of the test and validation phase about usability of functionalities allowed us to identify problems, solutions, and determine the validity of the solution. Thus some improvements have been applied to reach the version presented in this paper. Some considerations are reported in this section.

The positioning of the QR codes was very important. They should be placed by doors at different heights to accommodate for different walking positions, such as crawling during smoky conditions. The dimensions of the QR codes should be 2 to 4 times the size shown in Figure 2, so that the QR code can be captured from 80cm away in presence of a significant level of smoke. Initially, the QR codes consisted of 7% redundancy, increased to 30% recently. They are more robust due to lack of visibility and corruption [16].

When cellular signal levels are low, it is impossible to make voice phone calls for emergency communication, but the Mobile Emergency app was able to send the emergency alarm and receive instructions. The possibility of having a guided form (Figure 3) to communicate the emergency was really appreciated, since there was the security of providing the right data and in short time. The noise of the alarm also disrupts normal voice communications, therefore the chat communication, direct production of the emergency form, and reception of information about the emergency on the phone was more efficient than direct voice calls. When the situation is complicated by lack of visibility, respiratory difficulty due to smoke, or tearful eyes, the usage of a mobile device and reading the maps on the walls becomes difficult. In those critical conditions, using the Mobile Emergency app is the best choice. The app can be very useful for coordinating the activities of medical personnel in emergency situations, but a strong coordination with the Central Station is needed as well. In these situations, the communication and the information recovered via the Mobile Emergency app can be more robust, more precise, and more reliable than any voice mechanisms.

The adoption of mobile emergency solution proved to be very effective in spotting the position and communicating the occurrence of an emergency to the Central Station, thus reducing the time needed to reach the collecting areas. In smoky conditions, the time to reach safety areas was faster for medical personnel using the app compared to those who did not have the app. Another very important positive factor was the opportunity of being informed about the emergency status in any place in the hospital, thus reducing panic from people who are not involved with the situation. This effectively avoids many irrelevant calls to the central server and congestion of the system.

This paper addressed the aspects of communication with the medical personnel formalizing the modalities and the information to guide such personnel during emergency conditions with the support given by a Central Station, providing: information, emergency status, exits, link to responsible colleagues, directions to the collecting areas, guidelines, and dosages. The Mobile Emergency app is a proven solution, demonstrated in Careggi Hospital, the largest medical infrastructure in Florence and Tuscany area in Italy. Our app can help the hospital personnel to communicate with the in-hospital emergency headquarter (in the same emergency room) which can then provide the emergency medical personnel with better information to assure the best available care. We have identified the requirements, scenarios, and a mobile app that can efficiently cope with emergencies within hospitals. The Mobile Emergency app allowed medical personnel to be able to locate their position within large facilities, the site of emergency, location of exits, to get updated information about the emergency, and to push direct calls to the Central Station to appropriately handle the emergency situation.

The proposed solution is presently under trial by the Maxi Emergency team of the Careggi Hospital. The trial implies the usage of the solution during the exercitations for the master in emergency to pass successively for regular usage when this phase has been successfully completed.

## Abbreviations

UML: unified modeling language
PHP MySQL: PHP: Hypertext Preprocessor My Structured Query Language
PDA: personal device assistant
UMTS: universal mobile telecommunications system
GPRS: general packet radio service
QR: quick response
